# INTEREST GROUP SESSION—RESEARCH IN QUALITY OF CARE: LIVING IN AND LEAVING NURSING HOMES: THE FACTORS THAT CONTRIBUTE TO QUALITY OF LIFE, HEALTH, AND SAFETY OUTCOMES

**DOI:** 10.1093/geroni/igz038.1307

**Published:** 2019-11-08

**Authors:** Nancy Kusmaul, Mercedes Bern-Klug

**Affiliations:** 1 University of Maryland, Baltimore County, Baltimore, Maryland, United States; 2 University of Iowa, Iowa City, Iowa, Andorra

## Abstract

Nursing homes house some of the most vulnerable older adults. They often have complex medical conditions and/or cognitive impairments that put them at risk for negative outcomes and poor quality of life. These outcomes can be altered through incorporating evidence-based practices aimed to improve care and residents’ life experiences. In this symposium we will explore factors that are shown to influence outcomes and quality of life for people that live in and are discharged from, long term care settings. Amy Roberts and colleagues will explore the influences of nursing home social service staff qualifications on residents’ discharge outcomes. Colleen Galambos and colleagues will present findings on advance directives and their impact on reducing potentially avoidable hospitalizations. Kelsey Simons and colleagues will discuss the potential for unmet needs for mental health services as part of nursing home care transitions, and will discuss a model of quality improvement that addresses this gap in care. Vivian Miller will present findings on the impact transportation access has on the ability of community-dwelling family members to visit and provide social support to their family member residents in long-term care. Finally, Nancy Kusmaul and Gretchen Tucker report the findings of their study comparing perceptions of nursing home residents, direct care staff, management, and families on the care practices that influence resident health and quality of life while they live in a long term care setting.

